# Correlations in the EPR State Observables

**DOI:** 10.3390/e26060476

**Published:** 2024-05-30

**Authors:** Daniel F. Orsini, Luna R. N. Oliveira, Marcos G. E. da Luz

**Affiliations:** Departamento de Física, Universidade Federal do Paraná, Curitiba 81531-980, Brazil; dan.orsini@gmail.com (D.F.O.); lunaoliveira@ufpr.br (L.R.N.O.)

**Keywords:** quantum correlations, EPR states, entanglement, Bell’s inequalities, CHSH correlation

## Abstract

The identification and physical interpretation of arbitrary quantum correlations are not always effortless. Two features that can significantly influence the dispersion of the joint observable outcomes in a quantum bipartite system composed of systems I and II are: (a) All possible pairs of observables describing the composite are equally probable upon measurement, and (b) The absence of concurrence (positive reinforcement) between any of the observables within a particular system; implying that their associated operators do not commute. The so-called EPR states are known to observe (a). Here, we demonstrate in very general (but straightforward) terms that they also satisfy condition (b), a relevant technical fact often overlooked. As an illustration, we work out in detail the three-level systems, i.e., qutrits. Furthermore, given the special characteristics of EPR states (such as maximal entanglement, among others), one might intuitively expect the CHSH correlation, computed exclusively for the observables of qubit EPR states, to yield values greater than two, thereby violating Bell’s inequality. We show such a prediction does not hold true. In fact, the combined properties of (a) and (b) lead to a more limited range of values for the CHSH measure, not surpassing the nonlocality threshold of two. The present constitutes an instructive example of the subtleties of quantum correlations.

## 1. Introduction

Probability, as formally viewed in stochastic processes (see, e.g., [1]), is a fundamental ingredient for understanding countless natural phenomena. For instance, it is ubiquitous in the general framework of classical statistical mechanics. Probability is also a keystone in quantum mechanics, where the concept of probability amplitudes relates to the distribution of outcomes upon measurements. However, there are fundamental distinctions between the concept of randomness [2] in classical and quantum physics since probability can have a contrasting character in these two realms [3]. These differences are particularly noticeable in the correlation functions of physical quantities, i.e., observables [2,4].

Although many restrictions apply [5], correlations can be used as measures of the degree of determinism/randomness in a system. Moreover, certain situations might be relatively easy to pinpoint. Indeed, on the one hand, if there is a well-behaved mapping between *a* and *b*, with *a* and *b* possible values for *bona fide* observables *A* and *B* describing a problem (we are obviously not considering logical/philosophical digressions, like A= “the sun raises every morning” and B= “humans are mortal”, such that *A* and *B* are true, but with no causal association between *A* and *B*), e.g., θ and *r* in a classical Keplerian orbit, the correlation is “perfect”, and there should be a fully deterministic relation between them. On the other hand, if a specific value for *A* determines a range of allowable values for *B* as well as their frequencies of occurrence (with the same being true for *A* regarding *B*), this would indicate a stochastic connection between *A* and *B*. So, at least in principle, one could infer a joint probability for *A* and *B*, allowing one to define a proper correlation function for these observables, which we represent by c(a,b).

**Figure 1:**
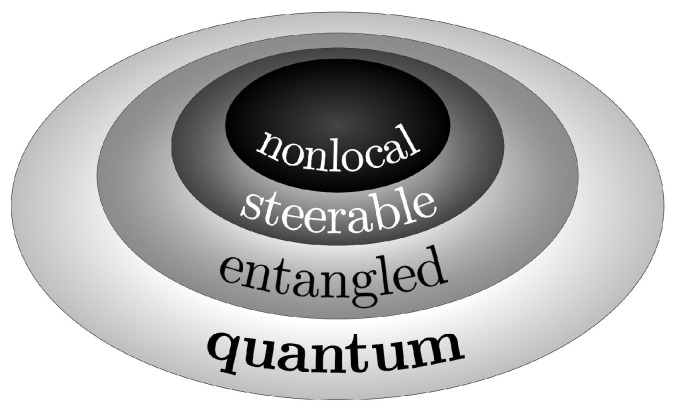
Schematics of the hierarchy of quantum correlations (see, e.g., [9]). However, there may be overlaps, which are not represented here. The basic one, simply ‘quantum’, relates to the very construction of quantum mechanics. It arises from superposition allied to interference: the probability density function p(q) is obtained from ${\left|\langle q|\psi \rangle \right|}^{2}$ with $|\psi \rangle =\sum_{n}c_{n}\;|\phi_{n}\rangle$, so that the “parts” $|\phi_{n}\rangle$s, whatever they represent, generate correlations. Beyond the basics, entanglement is typically considered the most fundamental form of quantum correlation, with nonlocality being the most restrictive. Steering falls in between, serving as a middle ground.

But as already mentioned, c will display different properties if resulting from either classical or quantum processes. We should mention that there are some general ways to identify between classical and quantum correlations, e.g., through the idea of distance measures as relative entropy [6]. Other procedures may be problem-oriented, for instance, those employed in the study of Gaussian states [7] or of system–reservoir interactions [8]. Quantum correlations are often more general than classical correlations [4,9,10], and in some cases stronger [11,12,13], creating scalings (due to entanglements) in many-body systems, which are classically absent [14,15]. Furthermore, there are distinct types of quantum correlations [9], ranging from the most basic—associated with the quantum construction itself, which we call “quantum”—to those exhibiting an increasing order of restrictiveness, typically entanglement, steering, and nonlocality; see Figure 1. Although the aim here is not to provide a general account of quantum correlations, it is useful for our later discussions to briefly mention a few characteristics of some of them. Also, since we shall specifically address EPR states (see Section 2), we restrict our analysis to pure states.

The first paradigmatic quantum correlation is entanglement, which includes all possible forms of interrelations in multipartite states. Focusing on the bipartite case, consider a composite state, $|\psi \rangle \in H=H_{I}⊗H_{II}$, where $H_{I}$ and $H_{II}$ are the Hilbert spaces of systems I and II; if this state *cannot* be written as the direct product ${|\mu \rangle }_{I}⊗{|\nu \rangle }_{II}$, for ${|\mu \rangle }_{I}\in H_{I}$ and ${|\nu \rangle }_{II}\in H_{II}$, then |ψ〉 is entangled. From this broad definition, one can have distinct degrees of entanglement for |ψ〉. Nonetheless, the entanglement of |ψ〉 is maximum if its von Neumann (or if one prefers, Shannon) entropy is also maximum. EPR states exactly meet this condition [16]. A second, somewhat stronger, coupling between systems I and II is that in which the state of one (e.g., I) can be driven or steered through measurements on the other (e.g., II); nevertheless, the contrary is not true. In such an inseparability context, the composite system exhibits a steering correlation (reviews in [9,17,18]). All steered systems are entangled, but not all entangled systems are steered. And third, suppose we measure the observable *A* for system II and *C* for system I (with I and II spatially apart), obtaining, respectively, *a* and *c*. In quantum mechanics, one finds that usually the joint probability p(a,c|A,C)≠p(a|A)p(c|C). The reason is that the quantum world is non-local—a notion heavily criticized by the famous EPR (Einstein–Podolsky–Rosen) paper [19] in the early days of the theory. An alternative interpretation would be to assume that quantum mechanics is incomplete, i.e., there exist local hidden variables λ inaccessible through its framework. In Bell’s groundbreaking contribution [20], it has been demonstrated that p(a,c|A,C)=∫dλp(λ)p(a|A,λ)p(c|C,λ) and some inequalities for the associated correlations should be observed. Nonetheless, the local hidden variables have been ruled out through experiments [21,22,23,24,25,26,27,28,29,30] showing violations of Bell’s inequalities. Thus, in opposition to EPR’s concept of local realism, nonlocality is inherent to quantum mechanics and indeed represents its most restrictive correlation [9,31]. We finally mention that any entangled pure state is non-local in some appropriately chosen set of observable bases, but the situation is far more involved for mixed states [9]; see also later. This previous (rather heuristic) hierarchical classification for quantum correlations is pictorially represented in Figure 1.

**Figure 2:**
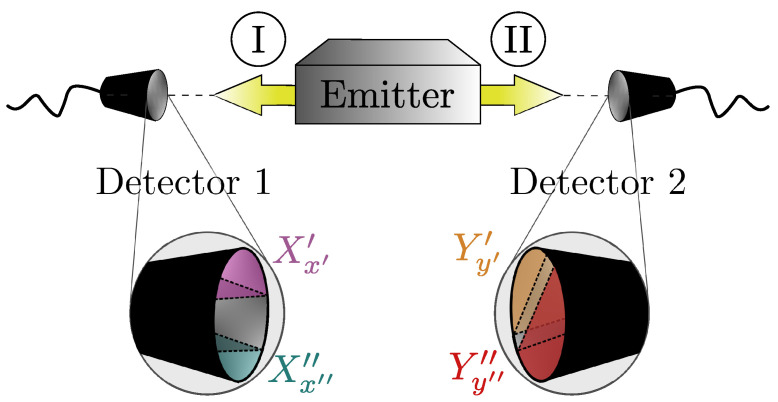
Illustration of the setup to compute the CHSH correlation function $c_{CHSH}$. By separating systems I and II, one can perform measurements of distinct observables, e.g., $X^{\prime }$ and $X^{\prime \prime }$ for I at detector 1 and $Y^{\prime }$ and $Y^{\prime \prime }$ for II at detector 2. From numerous realizations of the experiment, leading to distinct outcomes, $x^{\prime }$, $x^{\prime \prime }$, $y^{\prime }$, $y^{\prime \prime }$ (assuming only the values −1 or +1, in two-level systems), one obtains the correlation function $c_{CHSH}$ (the actual expression is given in Section 5).

As a significant example of Bell’s inequality for two-level systems (qubits), we refer to the CHSH inequality [32]. For an ensemble of composites formed by systems I and II, suppose that for all copies, parts I and II are spatially separated and then placed in regions 1 and 2, respectively. This should be done without altering the original properties that we intend to measure. So, detectors 1 and 2 in these regions can infer certain observables for I ($X^{\prime }$ and $X^{\prime \prime }$, whose results are denoted as $x^{\prime }$ and $x^{\prime \prime }$) and II ($Y^{\prime }$ and $Y^{\prime \prime }$, of results $y^{\prime }$ and $y^{\prime \prime }$); Figure 2. From appropriate averages, we can calculate the CHSH correlation function $c_{CHSH}\left(x^{\prime },x^{\prime \prime },y^{\prime },y^{\prime \prime }\right)$; for an explicit expression, see Section 5, and for an overview of the whole procedure, refer to [16]. For arbitrary *X* and *Y* observables, local hidden variables would necessarily lead to $c_{CHSH}\leq 2$. But quantum mechanics does allow situations where $c_{CHSH}>2$, corresponding to Bell’s inequality violation.

The above considerations might give the incorrect impression that identifying and classifying the quantum correlations of any state is straightforward. Actually, from a technical point of view this is often not true [9,33,34,35]. Moreover, determining all states that comply with certain specified correlation traits can be even harder [36]. One potential issue is that the interpretation of quantum correlations may contradict our intuitive understanding of classical correlations [2,4].

In this contribution, we address two concrete features for the observables of a bipartite state. Suppose we have a collection of observables $E^{\left(j\right)}$ for I and $F^{\left(j\right)}$ for II, with j=1,2,…,J. For any *j*, we assume that the allowed outcomes are always drawn from the same set of numerical values, respectively, $e^{\left(j\right)}=\left\{e_{1},e_{2},\ldots ,e_{N}\right\}$ and $f^{\left(j\right)}=\left\{f_{1},f_{2},\ldots ,f_{N}\right\}$. Notice that this is not a too-restrictive assumption. For instance, if the observables are the spin-1/2 components $S_{x}$, $S_{y}$, $S_{z}$, then the possible numerical values of measurements are always the same, i.e., ±ℏ/2. Also, let c be a quantum correlation function for these possible observables, taken in pairs ${\left(e_{n},f_{n}\right)}^{\left(j\right)}$. With the exception of potentially overly specific functional forms for c, typically, we can anticipate that larger (smaller) values for c are associated with a stronger (weaker) interdependence between $\left\{E^{\left(j\right)},F^{\left(j\right)}\right\}$. On the other hand, the range of variation for c should be connected to the particular relations within $\left\{E^{\left(j\right)}\right\}$ and within $\left\{F^{\left(j\right)}\right\}$. Thus, we consider two, in principle, independent conditions: (a) Regardless of *j*, only pairs in the form ${\left(e_{n},f_{n}\right)}^{\left(j\right)}$ can describe the composite system state. So, by determining the value of $E^{\left(j\right)}$ ($F^{\left(j\right)}$), we ascertain the value of $F^{\left(j\right)}$ ($E^{\left(j\right)}$). Furthermore, they are equally probable, i.e., we have a uniform distribution for these values, so that in an ensemble description, any pair, *n*, would contribute with the same probability, $p_{n}=1/N$. Hence, for $S^{\left(j\right)}=-\sum_{n}p_{n}\ln\left[p_{n}\right]$, and fixed *N*, this premise maximizes the normalized entropy $S^{\left(j\right)}/N=\ln\left[N\right]/N$ (note that $S^{\left(j\right)}/N$ is well-defined even in the hypothetical limit of N→∞). This represents the maximum entanglement of the composite. (b) There is no reinforcement dependence among $e^{\left(j\right)}$s or $f^{\left(j\right)}$s. On the contrary, knowing the value of $e^{\left(j^{\prime \prime }\right)}$ ($f^{\left(j^{\prime \prime }\right)}$) precludes knowing with certainty the value of $e^{\left(j^{\prime }\right)}$ ($f^{\left(j^{\prime }\right)}$), $j^{\prime \prime }\neq j^{\prime }$. This naturally emerges from the non-commutation, $[{\hat{E}}^{\left(j^{\prime \prime }\right)},{\hat{E}}^{\left(j^{\prime }\right)}]\neq 0$ and $[{\hat{F}}^{\left(j^{\prime \prime }\right)},{\hat{F}}^{\left(j^{\prime }\right)}]\neq 0$, of the corresponding self-adjoint operators for the observables $\left\{E^{\left(j\right)}\right\}$ and $\left\{F^{\left(j\right)}\right\}$.

Observe that (a) represents a substantial link between systems I and II. Indeed, knowing an observable in one system entails complete knowledge of the corresponding observable (because of the pairing ${\left(e_{n},f_{n}\right)}^{\left(j\right)}$) in the other. Also, we recall that for arbitrary entangled states, it is always possible to find suitable sets of observable bases, such that Bell’s inequalities are violated [9,37,38]. Hence, solely assuming (a), one might be led to conclude that $\left\{E^{\left(j\right)}\right\}$ and $\left\{F^{\left(j\right)}\right\}$ constitute these bases. Consequently, for N=2, taking c as $c_{CHSH}$ and supposing all possible combinations between $\left\{E^{\left(j^{\prime }\right)},F^{\left(j^{\prime }\right)}\right\}$ and $\left\{E^{\left(j^{\prime \prime }\right)},F^{\left(j^{\prime \prime }\right)}\right\}$ (e.g., representing the different choices of $X^{\prime }$, $X^{\prime \prime }$, $Y^{\prime }$, and $Y^{\prime \prime }$ in Figure 2), we would expect the non-observance of the Bell’s inequality, at least in some instances. On the contrary, considered alone, the condition (b) seems to act in a different direction, tending to decrease the quantum correlations. In this way, two pertinent queries arise: (i) Possible contexts where (a) and (b) occur together and (ii) given so, which values c can assume.

The arguments in [19] criticizing the nonlocality of quantum mechanics were fully based on particular pure bipartite states, the so-called EPR states |Ψ〉 — for a very complete and solid refutation of the EPR reasoning, demonstrating its inadequacy, see, e.g., [39] and the refs. therein. Interestingly, these |Ψ〉s verify the assumption (a) (as well as a second extra feature; see the next section). The conclusions in [19], and indirectly the structures of these |Ψ〉s, motivated the investigations in [20] (also refer to [40,41]). But as mentioned earlier, local realism has been overturned by testing Bell’s inequalities for arbitrary |ψ〉s.

Here, we prove very generally—and without the need for sophisticated mathematical techniques—that |Ψ〉s also comply with the assumption (b), thus, providing a concrete example of (i). In addition, as for (ii), setting N=2 (qubits), we calculate $c_{CHSH}$ considering only observables associated with EPR states. We determine that $c_{CHSH}\leq 2$; thus, not exceeding the nonlocality threshold of 2. It is worth recalling that maximally entangled pure states do not need to map into maximum violation of the CHSH inequality [42,43] (a fact motivating the proposal of distinct Bell-like inequalities capable of detecting maximal entanglement; see [44,45]). Actually, some maximally entangled pure states can adhere to the CHSH inequality under special local measurements. So, another result in the present contribution is that this is also always the case across all EPR bases.

This paper is organized as follows: We review important aspects of EPR states in Section 2. We give a simple demonstration of condition (b) for EPR states in Section 3. We exemplify our results for the three-level systems in Section 4. We show that EPR states do not violate Bell’s inequalities for CHSH correlations in Section 5. Our final remarks and conclusions are drawn in Section 6. For the sake of completeness, known but here very systematized facts about the concept of observables and features of observables bases and bases transformations are presented in the appendices.

## 2. Some Key Aspects in Forming and Measuring EPR States

In this Section we briefly review the basic characteristics of EPR states |Ψ〉 [46], essentially those considered in the original work by Einstein–Podolsky–Rosen [19]. A much more general and rigorous definition of finite dimensional systems can be found in [47,48] (for infinite-dimensional spaces, see [49,50]).

**Figure 3:**
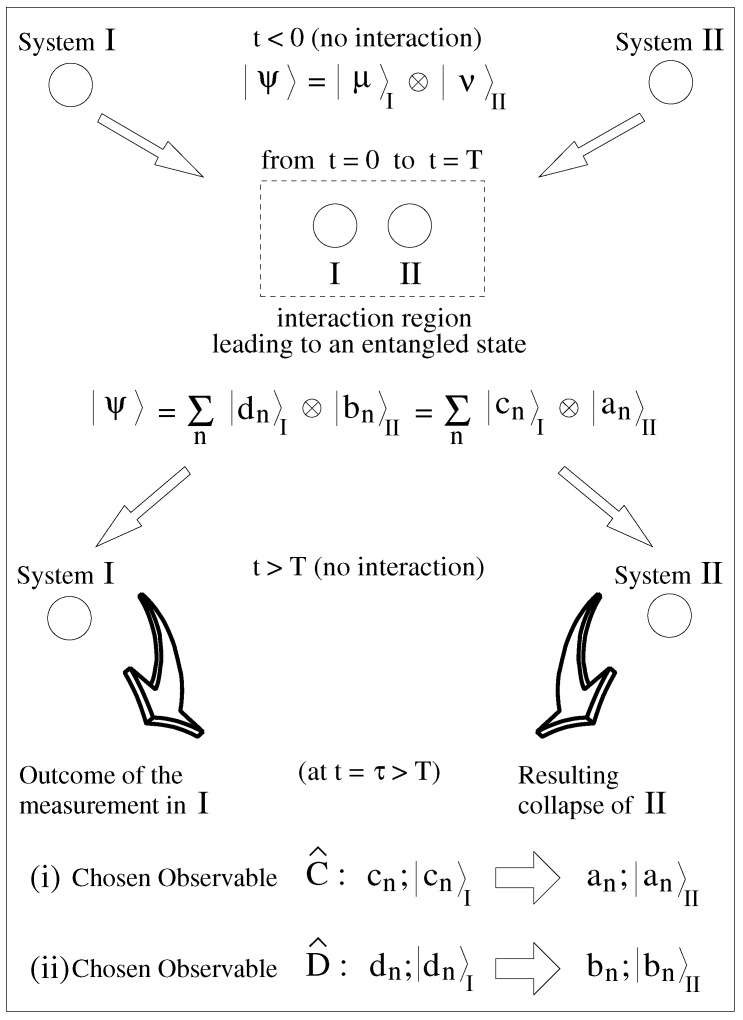
Schematics of typical processes in forming and measuring an EPR state |Ψ〉 (constituted by the entanglement of two systems, I and II): the preparation of the entangled state; the spatial separation of its two parts, I and II; and the possible measurements of an observable for system I, allowing the inference (with 100% certainty) of an observable for system II.

The main relevant steps in the preparation and posterior measurements of |Ψ〉 are schematically depicted in Figure 3. Initially (t<0), one has two non-interacting systems (systems I and II), with their composite state |Ψ(t<0)〉 simply being the direct product of the individual states of I and II. Then, during the time interval 0≤t≤T, systems I and II interact in such a way that certain physical observables of I (say *C*, associated with the Hermitian operator $\hat{C}$ having eigenvalues $\left\{c_{n}\right\}$, for n=1,2,…,N) and some of II (say *A*, associated with $\hat{A}$ of eigenvalues $\left\{a_{n}\right\}$) become entangled, resulting in the state |Ψ(T)〉. Next, during a time interval Δt, I and II are sufficiently brought apart so to cease any eventual influence of one another. Since the exact value of Δt is not relevant to the present discussion, for simplicity we just set Δt=0. Here, by influence we mean the type of interaction described by potentials ${\hat{V}}_{I–II}$ in the Schrödinger equation, coupling the degrees of freedom of systems I and II. Hence, for t>T, one has that ${\hat{V}}_{I–II}=0$. Moreover, one must guarantee that regardless of the separation procedure and the subsequent dynamics (for t>T), the entanglement attained between systems I and II (during 0≤t≤T) is maintained. This should hold until any eventual measurement at any t=τ>T. In this way, for T<t<τ, the components of |Ψ〉 corresponding to the eigenvectors $\left\{|c_{n}\rangle \right\}$ of I and $\left\{|a_{n}\rangle \right\}$ of II would not be altered, or

(1) $$|\Psi \left(t\right)\rangle =\sum_{n}\;|{\tilde{\mu }}_{n}\left(t\right),c_{n}\rangle_{I}⊗{|{\tilde{\nu }}_{n}\left(t\right),a_{n}\rangle }_{II}.$$

In Equation (1), the labels $\tilde{\mu }$ (for I) and $\tilde{\nu }$ (for II) denote how other observables, distinctly from *C* of I and *A* of II, will evolve in time, given that the systems I and II no longer interact.

If, by means of measurements, we aim to assess only the correlated quantities *C* and *A*, the information given by ${\tilde{\mu }}_{n}\left(t\right)$ and ${\tilde{\nu }}_{n}\left(t\right)$ is not fundamental. Thus, one can drop these explicit dependencies in Equation (1), just written it as $|\Psi \rangle =\sum_{n}\;u_{n}\;|c_{n}\rangle_{I}⊗{|a_{n}\rangle }_{II}$ for $\sum_{n}{\left|u_{n}\right|}^{2}=1$. It is a common practice in the analysis of EPR states [46,47]: (1) To suppose maximally entangled states, i.e., all $|c_{n}\rangle_{i}⊗{|a_{n}\rangle }_{II}$ in |Ψ〉 being equally probable, namely, for any *n* to assume that $u_{n}=\exp\left[I\;\varphi_{n}\right]/\sqrt{N}$. (2) To disregard eventual relative phases $\varphi_{n}$ by trivially reincorporating them into the state’s definition, or $\exp\left[i\;\varphi_{n}\right]\;|c_{n}\rangle_{I}⊗|a_{n}\rangle_{II}\to |c_{n}\rangle_{I}⊗{|a_{n}\rangle }_{II}$. Thus, the existence (or not) of phases multiplying the eigenvectors is irrelevant to all our general results.

A second fundamental feature of an EPR state is that |Ψ〉 (here, for T<t<τ, i.e., prior to any measurement) can be written in the following distinct ways:

(2) $$|\Psi \rangle =\frac{1}{\sqrt{N}}\sum_{n}\;|c_{n}\rangle_{I}⊗|a_{n}\rangle_{II}=\frac{1}{\sqrt{N}}\sum_{n}\;|d_{n}\rangle_{I}⊗{|b_{n}\rangle }_{II},$$

where the set $\left\{|e_{n}\rangle \right\}$ (e=a,b,c,d) is composed by the eigenvectors of $\hat{E}$ ($\hat{E}=\hat{A},\hat{B},\hat{C},\hat{D}$), or

(3) $$\hat{E}|e_{n}\rangle =e_{n}|e_{n}\rangle .$$

From Equation (2), we have that from a measurement performed only on I at t=τ>T—determining the observable *C* (*D*)—we should obtain complete knowledge about the value of *A* (*B*) for II; Figure 3. In fact, if afterward (t>τ) we test system II for *A* (*B*), we will find the same previously inferred value. Paramount to our analysis is the fact that Equation (2) and, thus, the EPR state, complies with the condition (a) discussed in the introduction.

One last feature in defining an EPR state is to ascribe to the observables *A* and *B*—associated with system II, cf. Equation (2)—operators that do not commute, i.e., $[\hat{A},\hat{B}]\neq 0$. In this respect, we mention that given an EPR state, one interesting and sometimes challenging issue is to determine all associated pairs of non-commuting observables [48]. We further remark that $[\hat{A},\hat{B}]\neq 0$ is a key assumption in [19], attempting to show that quantum mechanics is incomplete (of course misguidedly; for a summary of many valid objections to the EPR arguments see [39]).

Below we derive an extra general property for |Ψ〉, arising from Equation (2) and $[\hat{A},\hat{B}]\neq 0$. We show that for the observables *C* and *D*: $[\hat{C},\hat{D}]\neq 0$. Considering the pairs of observables *A*, *B* for II and *C*, *D* for I, this is precisely the condition (b) in Section 1. Therefore, it is established that EPR states simultaneously obey (a) and (b).

## 3. A Necessary Condition for EPR States: The Observables Are Pair-Wisely Associated with Non-Commuting Operators

Hereafter, we suppose the Hilbert space for the composed systems (refer to Equation (1)) $H_{I–II}={\left(H_{\mu }⊗H\right)}_{I}⊗{\left(H_{\nu }⊗H\right)}_{II}$. Note that $H_{\mu }$ and $H_{\nu }$ distinguish I from II. Further, we assume that all specific aspects that we wish to determine for our individual systems, either I or II, are described by the proper separable Hilbert space H (of dimensions *N* and spanned by a countable, i.e., discrete, basis—see Equation (4)). We should observe that although EPR states are usually addressed for finite *N*s, which is also the case in this contribution, quantum correlations, including CHSH, and |Ψ〉s for countable infinite-dimensional (N→∞) systems are also analyzed in the literature, see, e.g., Refs. [51,52], as well as the general considerations in [47].

Moreover, the reasons for choosing countable Hilbert spaces are twofold. First, EPR-like states have been vital to test certain fundamental predictions of quantum mechanics, even motivating the development of the Bell inequalities [20,40]. Albeit some theoretical proposals [53] and devised experimental arrangements [54,55,56] are based on continuous variables (for a review, see [57]), historical breakthroughs [21,22,23,24,25,26] and recent loophole-free measurements [27,28,29,30,58] consider discrete observables. Second, the spectrum theorem for self-adjoint operators—relevant for determining the suitable basis for |Ψ〉—holds true very generally [59]. However, establishing solidly grounded properties of transformations (similar to those presented in Section 3.1) between continuous bases may require additional technicalities; this goes far beyond the scope of this contribution. For instance, for continuous bases associated with operators such as position and momentum, one should work with generalized eigenvectors in a rigged Hilbert space [60].

As quantum observables, we suppose linear self-adjoint—or Hermitian, the usual jargon in physics—operators, whose domains are the whole H (eventually, one could also consider linear subsets of H, which are dense in H if the self-adjoint operators are unbounded [61]; see Appendix A). For our goals, it is not necessary to explicitly address formal constructions of the join probability measures associated with any assessment of observables, e.g., as rigorously conducted in [47]. We adopt fairly well-established definitions (refer to [47]) that are consistent with actual procedures in concrete measurement realizations [21,22,23,24,25,26].

For $\left\{|a_{n}\rangle \right\}$ and $\left\{|b_{n}\rangle \right\}$, the sets of eigenvectors of $\hat{A}$ and $\hat{B}$ forming the orthonormal basis (ONB) of H, the basis change $\{|a_{n}\rangle \}↔\{|b_{n}\rangle \}$ reads as follows (which in fact is valid for *N* either finite or infinite):

(4) $$|a_{n}\rangle =\sum_{m=1}^{m=N}\;\Gamma_{mn}\;|b_{m}\rangle ,\;\;\;\;\;\;\;\;|b_{n}\rangle =\sum_{m=1}^{m=N}\;{\left(\Gamma^{†}\right)}_{mn}\;|a_{m}\rangle .$$

To derive the results of the next Sec., we will rely on some known properties of the unitary matrix Γ, dependent on the commutation relation of $\hat{A}$ and $\hat{B}$, as discussed in Appendix A. Considerations about appropriate self-adjoint operators $\hat{A}$ and $\hat{B}$, representing observables for EPR states, are reviewed in Appendix A.1.

### 3.1. Correlations in the Observables of EPR States

From the above and the remarks in Section 2, for systems I and II described by EPR states, as seen in Equation (2), we can focus on the “reduced” Hilbert space ${\left(H\right)}_{I}⊗{\left(H\right)}_{II}$. Also, for the self-adjoint operators $\hat{A}$, $\hat{B}$, $\hat{C}$, and $\hat{D}$ (see Equation (3)), we assume that the features described in Appendix A.2 and $\hat{C}$ and $\hat{D}$ ($\hat{A}$ and $\hat{B}$) act just on I (II). Thus, the set of eigenvectors $\left\{|c_{n}\rangle \right\}$ and $\left\{|d_{n}\rangle \right\}$, respectively, of $\hat{C}$ and $\hat{D}$, are suitable ONBs for $H_{I}$. The same is true for $\left\{|a_{n}\rangle \right\}$ and $\left\{|b_{n}\rangle \right\}$) (of $\hat{A}$ and $\hat{B}$) with respect to $H_{II}$. We also recall that $[\hat{A},\hat{B}]\neq 0$.

By inserting the second relation in Equation (4) into the second equality in Equation (2), we obtain the following:

(5) $$|\Psi \rangle =\frac{1}{\sqrt{N}}\;\sum_{m=1}^{m=N}\;|d_{m}\rangle_{I}⊗\sum_{n=1}^{n=N}{\left(\Gamma^{†}\right)}_{nm}\;|a_{n}\rangle_{II}=\frac{1}{\sqrt{N}}\;\sum_{n=1}^{n=N}\;\left(\sum_{m=1}^{m=N}\;{\left(\Gamma^{†}\right)}_{nm}\;{|d_{m}\rangle }_{I}\right)⊗{|a_{n}\rangle }_{II}.$$

Comparing Equation (5) with Equation (2), (recalling that $\left\{|a_{n}\rangle \right\}$ is an ONB), we must have the following:

(6) $$|c_{n}\rangle_{I}=\sum_{m=1}^{m=N}\;\Gamma_{mn}^{*}\;{|d_{m}\;\rangle }_{I}.$$

Hence, from Equation (6), we see that up to the complex conjugation of the elements of Γ (an operation that certainly does not change the matrix structural relations), the basis transformation $\{|c_{n}\rangle \}↔\{|d_{n}\rangle \}$ is fully akin to $\{|a_{n}\rangle \}↔\{|b_{n}\rangle \}$. In other words, Equation (6) has exactly the same functional form of the mapping between $\left\{|a_{n}\rangle \right\}$ and $\left\{|b_{n}\rangle \right\}$.

But the matrix Γ—hence also $\Gamma^{†}$—represents a change of basis associated with non-commuting operators, $\hat{A}$ and $\hat{B}$. Thence, according to Appendix A.2, Γ cannot be transformed into an identity, or more generally, in a permutation $P_{\pi }$, matrix. In Appendix A.3, we illustrate such universal facts considering the bases transformations of a spin-1/2 system in arbitrary directions. So, $\hat{C}$, $\hat{D}$ cannot commute; otherwise, it would be possible to find a basis where they are simultaneously diagonal, leading to Γ→1 (or $\Gamma \to P_{\pi }$), so a contradiction.

To summarize, if a state |Ψ〉 can be expanded as in Equation (2)—with the different bases related to the Hermitian operators $\hat{A}$, $\hat{B}$, $\hat{C}$, $\hat{D}$—and $[\hat{A},\hat{B}]\neq 0$, we conclude that $[\hat{C},\hat{D}]\neq 0$. This is implied in our aforementioned condition (b). As far as we know, this property of the EPR state has gone somewhat unexplored in the literature, even though it is implicit in particular contexts, like in the Bohm construction of EPR states [62], in Bohr’s response [63] to the EPR paper [19], and in certain constructive empiricism critics to EPR reasoning [64].

## 4. An Explicit Example: Three-Level System

To illustrate the previous general result, we discuss qutrits, i.e., N=3. In this case, I and II can be described as three components of angular moment systems. Then, the equivalent to the Pauli matrices are (for the usual direction association: 1 for *x*, 2 for *y*, 3 for *z*)

(7) $$\sigma_{1}=\frac{1}{\sqrt{2}}\left(\begin{array}{ccc} 0 & +1 & 0\\ +1 & 0 & +1\\ 0 & +1 & 0 \end{array}\right),\;\;\sigma_{2}=\frac{1}{\sqrt{2}}\left(\begin{array}{ccc} 0 & -i & 0\\ +i & \;0 & -i\\ 0 & \;+i & 0 \end{array}\right),\;\;\sigma_{3}=\left(\begin{array}{ccc} +1 & \;0 & 0\\ 0 & \;0 & 0\\ 0 & \;0 & -1 \end{array}\right).$$

Note that $\left[\sigma_{u},\sigma_{v}\right]=i\;\sum_{w=-1}^{+1}\;\epsilon_{uvw}\;\sigma_{w}$ for $\epsilon_{uvw}$ the permutation symbol of u,v,w. The eigenvalue equation associated with $\sigma_{w}$ (with w=1,2,3) reads $\sigma_{w}\;|w,m\rangle =m\;|w,m\rangle$, where m=+1,0,−1. One has

(8) $$\begin{array}{ccc} |3,+1\rangle =\left(\begin{array}{c} 1\\ 0\\ 0 \end{array}\right), & |3,0\rangle =\left(\begin{array}{c} 0\\ 1\\ 0 \end{array}\right), & |3,-1\rangle =\left(\begin{array}{c} 0\\ 0\\ 1 \end{array}\right),\\ |2,+1\rangle =\frac{1}{2}\left(\begin{array}{c} -1\\ -i\sqrt{2}\\ 1 \end{array}\right), & |2,0\rangle =\frac{1}{2}\left(\begin{array}{c} \sqrt{2}\\ 0\\ \sqrt{2} \end{array}\right), & |2,-1\rangle =\frac{1}{2}\left(\begin{array}{c} -1\\ i\sqrt{2}\\ 1 \end{array}\right),\\ |1,+1\rangle =\frac{1}{2}\left(\begin{array}{c} 1\\ \sqrt{2}\\ 1 \end{array}\right), & |1,0\rangle =\frac{1}{2}\left(\begin{array}{c} -\sqrt{2}\\ 0\\ \sqrt{2} \end{array}\right), & |1,-1\rangle =\frac{1}{2}\left(\begin{array}{c} 1\\ -\sqrt{2}\\ 1 \end{array}\right). \end{array}$$

Just as a mathematical digression, we observe all the above eigenvectors can be condensed into a single formula as follows:

(9) $$|w,m\rangle =\frac{\sqrt{2-|m|}}{2}\left\{\left(\begin{array}{c} {\left(-1\right)}^{w+m}\\ {\left(-i\right)}^{w-1}\;\sqrt{2}\;m\\ 1 \end{array}\right)+\delta_{w\;3}\;\left(m+\delta_{m\;0}\right)\;\left(\begin{array}{c} 1\\ \sqrt{2}\\ -1 \end{array}\right)\right\}.$$

From the above expressions, we can readily construct the basis transformation matrices $\Gamma^{v\to w}$, such that $|w,m\rangle =\sum_{n=-1}^{+1}\Gamma_{n\;m}^{\left(v\to w\right)}|v,n\rangle$. For example, $\Gamma^{3\to 1}$ is given by the following:

(10) $$\begin{array}{ccc} \Gamma^{\left(3\to 1\right)} & = & \frac{1}{2}\left(\begin{array}{ccc} 1 & -\sqrt{2} & 1\\ \sqrt{2} & 0 & -\sqrt{2}\\ 1 & \sqrt{2} & 1 \end{array}\right). \end{array}$$

Furthermore, in a compact form we can write the following:

(11) $$\Gamma^{\left(3\to w=1,2\right)}=\frac{1}{2}\left(\begin{array}{ccc} {\left(-1\right)}^{w-1} & {\left(-1\right)}^{w}\;\sqrt{2} & {\left(-1\right)}^{w-1}\\ {\left(-i\right)}^{w-1}\;\sqrt{2} & \;0\; & -{\left(-i\right)}^{w-1}\;\sqrt{2}\\ 1 & \;\sqrt{2}\; & 1 \end{array}\right)$$

and

(12) $$\Gamma^{\left(v=1,2\to w=2,1\right)}=\frac{1}{2}\left(\begin{array}{ccc} (v-w)\;i & \;\sqrt{2}\; & (w-v)\;i\\ \sqrt{2} & \;0\; & \sqrt{2}\\ (w-v)\;i & \;\sqrt{2}\; & (v-w)\;i \end{array}\right).$$

Also, the matrices $\Gamma^{\left(1,2\to 3\right)}$ and $\Gamma^{\left(2,1\to 1,2\right)}$ follow from $\Gamma^{\left(w\to v\right)}={\Gamma^{\left(v\to w\right)}}^{†}$ (obviously, $\Gamma^{\left(w\to w\right)}=1$).

In Table 1, we show that regardless of *w*, *v*, *u*, and *m*, we will always have the following:

(13) $$\sum_{n=-1}^{+1}{\Gamma_{nm}^{\left(u\to v\right)}}^{*}|w,n\rangle ={\left(i\right)}^{k}\;|t,{\left(-1\right)}^{l}\;m\rangle ,$$

where k=0,1,2,3; l=0,1 and t=1,2,3, but with the rather important restriction, as follows:

(14) t≠w.

Next, by constructing EPR states for three-level systems, we show that as it should be, obligatorily the observables need to be associated with non-commuting operators. We start with (for w,u=1,2,3 totally arbitrary), as follows:

(15) $$\begin{array}{ccc} |\Phi \rangle & = & \frac{1}{\sqrt{3}}\sum_{n}{|w,n\rangle }_{I}⊗{|u,n\rangle }_{II}. \end{array}$$

For any v=1,2,3, and using the properties of the transformation matrices Γ, for Equation (15) we have the following:

(16) $$|\Phi \rangle =\frac{1}{\sqrt{3}}\sum_{n}{|w,n\rangle }_{I}⊗\left(\sum_{m}\Gamma_{mn}^{\left(v\to u\right)}{|v,m\rangle }_{II}\right)=\frac{1}{\sqrt{3}}\sum_{m}\left(\sum_{n}{\Gamma_{nm}^{\left(u\to v\right)}}^{*}{|w,n\rangle }_{I}\right)⊗{|v,m\rangle }_{II}.$$

For u≠v, the operators $\sigma_{u,\;II}$ and $\sigma_{v,\;II}$ for system II do not commute. We recall that this is one of the requirements of EPR states. Moreover, in such a case, due to Equation (13) and Table 1, we have that $\sum_{n}{\Gamma_{nm}^{\left(u\to v\right)}}^{*}{|w,n\rangle }_{I}$—see the last equality in Equation (16)—is necessarily one of the eigenvectors of the operator $\sigma_{t,\;I}$ (eventually multiplied by a phase $I^{k}$). Also, according to the condition in Equation (14), $\sigma_{t,\;I}$ does not commute with $\sigma_{w,\;I}$. So, this is in agreement with condition (b) of the EPR states proved in the previous section. Lastly, for *f* a one-to-one index function from {1,0,−1} to {1,0,−1} (easily inferred from Table 1) and $k_{m}$ integer numbers, we find very generally that (w≠t and u≠v)

(17) $$|\Phi \rangle =\frac{1}{\sqrt{3}}\sum_{m}{|w,m\rangle }_{I}⊗{|u,m\rangle }_{II}=\frac{1}{\sqrt{3}}\sum_{m}i^{k_{m}}{\;|t,f\left(m\right)\rangle }_{I}⊗{|v,m\rangle }_{II}.$$

In this way, |Φ〉 is an EPR state |Ψ〉, displaying all the necessary properties regarding the non-commutation of the associated observables, i.e., $[\sigma_{u,\;II},\sigma_{v,\;II}]\neq 0$ and $[\sigma_{w,\;I},\sigma_{t,\;I}]\neq 0$.

It should not be difficult to show that, using the above results, the same holds true if we construct EPR states from the eigenstates of the operators $\sigma_{\alpha ,\beta ,\gamma }=\alpha \;\sigma_{1}+\beta \;\sigma_{2}+\gamma \;\sigma_{3}$.

Finally, the findings in Section 3 follow irrespective of whether the states considered are degenerate or not. Hence, it is instructive to provide an example of the former situation. We note that, with the help of [65], it is straightforward to engender Hermitian matrices displaying degenerated eigenvalues. Thus, suppose the eigenvalues $\zeta_{m}=\left|1+2\;m\right|$ (or $\zeta_{1}=3$, $\zeta_{0}=\zeta_{-1}=1$) of the eigenvectors and associated observable operator:

(18) $$|\zeta_{m}\rangle =\frac{1}{\sqrt{1+|m|}}\;\left(\begin{array}{c} m\\ 1-|m|\\ \left|m\right| \end{array}\right),\;\sigma_{\zeta }=\left(\begin{array}{ccc} 2 & 0 & 1\\ 0 & 1 & 0\\ 1 & 0 & 2 \end{array}\right).$$

We set

(19) $$|\Omega \rangle =\frac{1}{\sqrt{3}}\sum_{m}|\zeta_{m}\rangle_{I}⊗{|1,m\rangle }_{II}.$$

Repeating the transformations implemented in Equation (16), i.e., changing the basis from ${|1,m\rangle }_{II}$ to ${|2,m\rangle }_{II}$ and considering the explicit form of ${\Gamma^{\left(1\to 2\right)}}^{*}$, we obtain (for f:{1,0,−1}↦{1,−1,0}, with $f\left(m\right)=-\left(m+1\right)/{\left(-2\right)}^{m}$)

(20) $$|\Omega \rangle =\frac{1}{\sqrt{3}}\sum_{m}|\eta_{f(m)}\rangle_{I}⊗{|2,m\rangle }_{II}.$$

Here, $\sigma_{\eta }\;|\eta_{m}\rangle =\eta_{m}\;|\eta_{m}\rangle$ with $\eta_{m}=\left|1+2\;m\right|$ (so $\eta_{1}=3$, $\eta_{0}=\eta_{-1}=1$) and

(21) $$|\eta_{m}\rangle =\frac{1}{\sqrt{1+\left(m+1\right)/2^{m}}}\;\left(\begin{array}{c} -i\;\left(m+1\right)/{\left(-2\right)}^{m}\\ \left(m+1\right)/2^{m}\\ m(m-1)/2 \end{array}\right),\;\sigma_{\eta }=\left(\begin{array}{ccc} 2 & +i & \;0\\ -i & 2 & \;0\\ 0 & 0 & \;1 \end{array}\right).$$

Then, from the matrices for $\sigma_{\zeta }$ and $\sigma_{\eta }$, it reads that $[\sigma_{\zeta ,\;I},\sigma_{\eta ,\;I}]\neq 0$ and given the exact form of Equations (19) and (20), as well as $[\sigma_{1,\;II},\sigma_{2,\;II}]\neq 0$, we conclude that |Ω〉 is an EPR state, thus satisfying (b).

## 5. Observables Exclusively Involving Qubits in EPR States Do Not Violate Bell Inequalities

As already mentioned, in principle, it is possible to find appropriate bases for an entangled state, such that $c_{CHSH}$ violates Bell’s inequality [9,37,38]. But referring to Figure 2, this implies properly choosing $X^{\prime }$, $X^{\prime \prime }$, and $Y^{\prime }$, $Y^{\prime \prime }$ to be measured, respectively, in detectors 1 and 2. A traditional example is that of entangled photons [16], where one can have the CHSH correlation $c_{CHSH}>2$ by adjusting the relative angles of the polarizers employed as detectors.

However, EPR states display a rather special link between their observables, adhering to conditions (a) and (b). Indeed, when considering Equation (2), on the one hand, if we know an observable value for system I (II), this fully determines the value of the associated observable for system II (I). On the other hand, observables in each system are pair-wisely incompatible, namely, we cannot simultaneously determine *A* and *B* for II and *C* and *D* for I. Conceivably, this must strongly affect the state’s correlation features. Hence, a pertinent question regards the possible range for $c_{CHSH}$ if computed only from EPR states. This seems to be a simple and direct way to characterize correlations for such a particular class of states.

Assuming the EPR-like state in Equation (2), let N=2 with the possible eigenvalues of $\hat{E}$$(=\;\;\hat{A},\hat{B},\hat{C},\hat{D}$) being ±1 (so $\hat{E}\;|e_{n}\rangle =\pm |e_{n}\rangle$, n=1,2). For simplicity, we disregard the eventual relative phases between the states, thus, we have the following:

(22) $$|\Psi \rangle =\frac{1}{\sqrt{2}}\left(|c_{1}\rangle_{I}⊗|a_{1}\rangle_{II}+|c_{2}\rangle_{I}⊗{|a_{2}\rangle }_{II}\right)=\frac{1}{\sqrt{2}}\left(|d_{1}\rangle_{I}⊗|b_{1}\rangle_{II}+|d_{2}\rangle_{I}⊗{|b_{2}\rangle }_{II}\right).$$

Nonetheless, we emphasize that writing $|c_{2}\rangle_{I}⊗|a_{2}\rangle_{II}\to \exp\left[i\mu \right]\;|c_{2}\rangle_{I}⊗{|a_{2}\rangle }_{II}$ and $|d_{2}\rangle_{I}⊗|b_{2}\rangle_{II}\to \exp\left[I\nu \right]\;|d_{2}\rangle_{I}⊗{|b_{2}\rangle }_{II}$ does not change the following results.

The transformation from the basis $\left\{|b_{n}\rangle \right\}$ to $\left\{|a_{n}\rangle \right\}$ is given by an arbitrary unitary matrix $\Gamma^{B\to A}$, whereas from $\left\{|d_{n}\rangle \right\}$ to $\left\{|c_{n}\rangle \right\}$ is given by the complex conjugate of $\Gamma^{B\to A}$ (cf., Equations (5) and (6)), or $\Gamma^{D\to C}={\Gamma^{B\to A}}^{†}$. Using an appropriate parameterization for U(2) matrices (see, e.g., [66]), we generally have

(23) $$\begin{array}{ccc} \Gamma^{B\to A} & = & \left(\begin{array}{cc} \alpha & \beta \\ -\beta^{*}\;\exp\left[i\theta \right] & \alpha^{*}\;\exp\left[i\theta \right] \end{array}\right), \end{array}$$

with α,β complex numbers such that ${\left|\alpha \right|}^{2}+{\left|\beta \right|}^{2}=1$ and 0≤θ<2π. Moreover, we suppose α,β≠0, so that $[\hat{A},\hat{B}]\neq 0$ (see Appendix A.3). Thus,

(24) $$\begin{array}{ccc} |a_{1}\rangle & = & \alpha \;|b_{1}\rangle -\beta^{*}\exp\left[i\theta \right]\;|b_{2}\rangle ,\\ |a_{2}\rangle & = & \beta \;|b_{1}\rangle +\alpha^{*}\exp\left[i\theta \right]\;|b_{2}\rangle ,\\ |b_{1}\rangle & = & \alpha^{*}\;|a_{1}\rangle +\beta^{*}\;|a_{2}\rangle ,\\ |b_{2}\rangle & = & -\beta \exp\left[-i\theta \right]\;|a_{1}\rangle +\alpha \exp\left[-i\theta \right]\;|a_{2}\rangle . \end{array}$$

Therefore, for the observables A,B,C,D, the CHSH correlation function $c_{CHSH}$ [32] reads as follows:

(25) $$c_{CHSH}=\left|\epsilon_{CA}-\epsilon_{CB}+\epsilon_{DA}+\epsilon_{DB}\right|,$$

with (see Figure 2)

(26) $$\epsilon_{XY}=\langle \Psi |{\hat{X}}_{I}⊗{\hat{Y}}_{II}|\Psi \rangle .$$

To obtain $\epsilon_{CA}$ (as well as $\epsilon_{CB}$) and $\epsilon_{DB}$ (as well as $\epsilon_{DA}$), we consider Equation (26) with |Ψ〉 given, respectively, by the first and second expansions in Equation (22). So, straightforwardly, we find the following:

(27) $$\epsilon_{CA}=\frac{1}{2}\left(c_{1}\;a_{1}+c_{2}\;a_{2}\right),\;\epsilon_{DB}=\frac{1}{2}\left(d_{1}\;b_{1}+d_{2}\;b_{2}\right).$$

For $\epsilon_{CB}$ and $\epsilon_{DA}$ we obtain the following:

(28) $$\epsilon_{CB}=\frac{1}{2}\left(c_{1}\;\langle a_{1}|\hat{B}|a_{1}\rangle +c_{2}\;\langle a_{2}|\hat{B}|a_{2}\rangle \right),\;\;\;\;\;\epsilon_{DA}=\frac{1}{2}\left(d_{1}\;\langle b_{1}|\hat{A}|b_{1}\rangle +d_{2}\;\langle b_{2}|\hat{A}|b_{2}\rangle \right).$$

Now, using Equation (24) in Equation (28)

(29) $$\begin{array}{ccc} \epsilon_{CB} & = & \frac{1}{2}\left(c_{1}\;(b_{1}{\;|\alpha |}^{2}+b_{2}{\;|\beta |}^{2})+c_{2}\;(b_{1}{\;|\beta |}^{2}+b_{2}{\;|\alpha |}^{2})\right),\\ \epsilon_{DA} & = & \frac{1}{2}\left(d_{1}\;(a_{1}{\;|\alpha |}^{2}+a_{2}{\;|\beta |}^{2})+d_{2}\;(a_{1}{\;|\beta |}^{2}+a_{2}{\;|\alpha |}^{2})\right). \end{array}$$

Finally (for ${\left|\alpha \right|}^{2}=1-\eta$, ${\left|\beta \right|}^{2}=\eta$, 0<η<1)

(30) $$\begin{array}{ccc} c_{CHSH} & = & \frac{1}{2}|\left(d_{1}+c_{1}\right)a_{1}+\left(d_{2}+c_{2}\right)a_{2}+\left(d_{1}-c_{1}\right)b_{1}+\left(d_{2}-c_{2}\right)b_{2}\\ \; & \; & +\eta (\left(c_{2}-c_{1}\right)\left(b_{2}-b_{1}\right)-\left(d_{2}-d_{1}\right)\left(a_{2}-a_{1}\right))|. \end{array}$$

Analyzing all the combinations (i.e., $e_{1}=+1$, $e_{2}=-1$ or $e_{1}=-1$, $e_{2}=+1$) for $e_{n}=a_{n}$, $b_{n}$, $c_{n},d_{n}$, one realizes that the only possibilities for $c_{CHSH}$ are

(31) $$c_{CHSH}=2\;or\;c_{CHSH}=2\;\left|2\eta -1\right|.$$

Hence, if we calculate $c_{CHSH}$ for any set of observables for which the state has the EPR structure, we always find that $c_{CHSH}\leq 2$. However, the violation of Bell’s inequalities in the CHSH construction corresponds to $c_{CHSH}>2$ (in fact, $2<c_{CHSH}\leq 2\;\sqrt{2}$ [67]).

The above finding is a bit curious. EPR states have been discussed in [19] as an attempt to show that quantum mechanics is incomplete, opening up the possibility of alternatives like local hidden variables. The violation of the Bell inequalities [20,40] for a quantum system overturns such claims [41]. Moreover, many breakthrough experiments have clearly demonstrated the existence of quantum correlations violating Bell’s inequalities [21,22,23,24,25,26,27,28,29,30]. However, when probing only the EPR observables (when N=2) in a CHSH-like experiment—without considering any additional information about the composite—one most likely would not be able to fully discard local hidden variables.

Also, the present is complementary to Hardy’s results [68,69], refuting local hidden variables without invoking inequalities. Indeed, he demonstrated Bell’s nonlocality through clever interference-like experimental setups for particle–antiparticle pairs. More recently, this idea has been extended to many particle systems [70].

## 6. Final Remarks and Conclusions

Quantum correlations are notoriously more diverse and conceptually more complex than their classical counterparts. For instance, certain works (see, e.g., [71]) argue that tests on violations of Bell-type inequalities should be interpreted as statistical inference of the local incompatibility of observables. So, they might deceive our intuitive predictions about trending behaviors. This is a direct consequence of the complex way in which observables are interrelated in certain particular states. Here, we have investigated one of these special |Ψ〉 states: bipartite EPR states.

EPR states are known to display maximum entanglement, in the sense that the determination of an observable for I (II) fully specifies the observable for II (I). Moreover, all pairs of observable values are equally probable. These characteristics constitute condition (a)—introduction section. In this contribution, through a rather simple procedure, we further established that, generally, if |Ψ〉 can be expanded in terms of eigenvectors, either for the observables *C* (of I) and *A* (of II) or *D* (of I) and *B* (of II), then *C* is incompatible with *D*, and *A* is incompatible with *B*. In other words, for the associated Hermitian operators, it follows that $[\hat{A},\hat{B}]\neq 0$ and $[\hat{C},\hat{D}]\neq 0$. This corresponds to condition (b). To illustrate this general result, we analyzed the EPR states in three-level systems (N=3).

From (a), there is a great interdependence between systems I and II: $c_{n}↔a_{n}$, $d_{n}↔b_{n}$, etc. But from (a) and (b), all possible pairs *n* ($\left(c_{n},a_{n}\right)$, $\left(d_{n},b_{n}\right)$, etc) of measurement outcomes are equiprobable and the observables within each system (*C* with *D*, *A* with *B*, etc.) are incompatible. This conceivably should lead to high fluctuations in the observable values, tending to decrease correlations. Combined, these opposite traits can make it difficult to foresee ranges for quantum cs obtained solely from the observables forming the |Ψ〉s. In the particular situation of qubits (N=2), by calculating $c_{CHSH}$ only for EPR states, we found that $c_{CHSH}\leq 2$, thus not violating Bell’s inequality.

Finally, a natural issue concerns the behavior of quantum correlations for EPR states in higher-dimensional bipartite systems, i.e., for N≥3. For such an analysis, it would be necessary to properly extend Bell’s inequalities, particularly for the CHSH. Some interesting proposals have already been addressed in the literature [72,73,74]. Nevertheless, irrespective of the exact analytic form of a generalized $c_{CHSH}$, the scheme should involve, as in Section 5, unitary matrices Γ of order N×N. Since a Γ∈ U(*N*) has $N^{2}$ free parameters, exact analytic calculations may be laborious, although not unfeasible (at least in some particular instances). It is our plan to discuss these and other aspects related to N≥3 in a future contribution.

## Figures and Tables

**Table 1:** List of all the states in the form ${\left(i\right)}^{k}\;|t,{\left(-1\right)}^{l}\;m\rangle$ (with k=0,1,2,3; l=0,1; t=1,2,3) resulting from $\sum_{n}{\Gamma_{nm}^{\left(u\to v\right)}}^{*}|w,n\rangle$, Equation (13), as one varies m=1,0,−1 and w,u,v=1,2,3.

	w=1, any *m*	w=2, any *m*	w=3, any *m*
${\Gamma^{\left(1\to 2\right)}}^{*}$	|2,−m〉	|1,m〉	${\left(-i\right)}^{m}\;|2,-m\rangle$
${\Gamma^{\left(2\to 1\right)}}^{*}$	|2,m〉	|1,−m〉	$i^{m}\;|2,m\rangle$
${\Gamma^{\left(1\to 3\right)}}^{*}$	|3,m〉	${\left(-i\right)}^{m+1}\;|3,m\rangle$	${\left(-1\right)}^{m+1}\;|1,-m\rangle$
${\Gamma^{\left(3\to 1\right)}}^{*}$	${\left(-1\right)}^{m+1}\;|3,-m\rangle$	$-{\left(-i\right)}^{m+1}\;|3,-m\rangle$	|1,m〉
${\Gamma^{\left(2\to 3\right)}}^{*}$	${\left(-i\right)}^{m+1}\;|3,m\rangle$	${\left(-1\right)}^{m+1}|3,m\rangle$	$i^{m+1}\;|1,-m\rangle$
${\Gamma^{\left(3\to 2\right)}}^{*}$	$i^{m}\;|2,-m\rangle$	$i^{m}\;|1,m\rangle$	|2,−m〉

## Data Availability

Data is contained within the article.

## Abbreviations

The following abbreviations are used in this manuscript:

EPR	Einstein-Podolsky-Rosen
CHSH	Clauser–Horne–Shimony–Holt
ONB	Orthonormal basis

## Appendix A. Transformations between Eigenbasis Associated with Commuting and Non-Commuting Operators

Below we review some important characteristics of observables and transformations between the countable basis of a separable Hilbert space H. These are all well known results, but here organized in a compact although comprehensive way.

We start by addressing the dimensions of the unitary Γ (i.e., $\Gamma \;\Gamma^{†}=\Gamma^{†}\;\Gamma =1_{N}$) in Equation (4). For finite *N*, the features of the transformation matrix are simply rooted in standard linear algebra. However, for the infinite case, the issue is whether the corresponding Γ is mathematically well-defined. In other words, one must ensure that typical matrix operations, such as multiplication, remain valid for N=∞ (at least to ensure unitarity).

The answer is positively provided: (i) We deal with suitable ONBs for H (see below); and (ii) The elements of Γ essentially represent inner products between basis eigenvectors, namely, $\Gamma_{mn}=\langle b_{m}|a_{n}\rangle$ and ${\left(\Gamma^{†}\right)}_{nm}=\langle a_{n}|b_{m}\rangle ={\langle b_{m}|a_{n}\rangle }^{*}={\left(\Gamma_{mn}\right)}^{*}$; refer to Equation (4). Then, Lemma 1.13 in [75] (see also [76]) guarantees that Γ, $\Gamma^{†}$, $\Gamma \;\Gamma^{†}$, and $\Gamma^{†}\;\Gamma$ are all meaningful and that Γ and $\Gamma^{†}$ in Equation (4) are unitary regardless of *N*. For further details, we cite thorough studies in the literature about infinite-by-infinite unitary matrices [77], the infinite matrix representation theory of operators in Hilbert spaces [78,79]—including the unbounded case [80]—and transformations between infinite ONBs [76,81]. We recall that for D(H) the domain of $\hat{A}$ in the Hilbert space H, the norm of $\hat{A}$, $\left|\right|\hat{A}\;\left|\right|$, is given by $\sup||\hat{A}\;|\psi \rangle \;||$ with |ψ〉∈D(H) and |||ψ〉||=1. $\hat{A}$ is said bounded (unbounded) if $\left|\right|\hat{A}\;\left|\right|$ is finite (infinite).

### Appendix A.1. The Observables Assumed for EPR States

For an EPR state, |Ψ〉, in a given Hilbert space, H, one key point is the specific ways |Ψ〉 can be expanded in terms of the eigenbasis of pertinent observables (cf., Equation (2)). Thence, an important question is whether or not this eigenbasis can properly span H. In the following, we summarize only certain facts about the *spectrum* $\sigma (\hat{A})$ of a general self-adjoint operator $\hat{A}$, relevant to settle the query. For a throughout account of this subject, please refer to [78,82,83] (and in this particular context of quantum mechanics, refer to [84,85,86]). A real $a\in \sigma (\hat{A})$ *iff* the resolvent ${\hat{R}}_{\hat{A}}\left(a\right)\equiv {\left(\hat{A}-a\;1\right)}^{-1}$ is not a bounded operator in the full domain of $\hat{A}$. There are different ways to classify $\sigma (\hat{A})$ (see, e.g., [82]). The one here—perhaps the most appropriate in quantum mechanics—is based on the Lebesgue decomposition of a measure on R [85]. So, $\sigma (\hat{A})$ is given by the union of the not necessarily disjoint sets $\sigma_{pp}\left(\hat{A}\right)$, $\sigma_{ac}\left(\hat{A}\right)$, $\sigma_{sc}\left(\hat{A}\right)$, respectively, representing the pure point, absolutely continuous, and singular continuous spectra. Vectors associated with each one of these sets (see, e.g., [78,82]) form three mutually orthogonal subspaces, $H_{pp}$, $H_{ac}$, and $H_{sc}$, all invariant under $\hat{A}$. Then, H can be written as the direct sum:

(A1) $$H=H_{pp}⊕H_{ac}⊕H_{sc}.$$

In standard quantum physics, the pure point and absolutely continuous parts of the spectrum are associated, respectively, with bound and scattering states [87,88,89]. Much less common is a non-empty $\sigma_{sc}$ [90,91,92], generally related to special Hamiltonians, e.g., with random or ‘bump’ [90] potentials. In such cases, the singular continuous spectrum accounts for phenomena like Anderson localization (see [88] and refs. therein).

In our present context, according to Equations (2) and (3), appropriate values for the observable *A* of an EPR state are the *a*s in the spectrum set $\sigma_{p}\left(\hat{A}\right)$. Indeed, the eigenvalue *a* belongs to $\sigma_{p}\left(\hat{A}\right)$ if there is at least one (eigenvector) |a〉 in the domain of $\hat{A}$ satisfying $\hat{A}\;|a\rangle =a\;|a\rangle$. Since $\sigma_{p}\left(\hat{A}\right)$ is a subset of $\sigma_{pp}\left(\hat{A}\right)$— note that $\sigma_{pp}\left(\hat{A}\right)$ is the closure of $\sigma_{p}\left(\hat{A}\right)$ [86], hence it follows that $\sigma_{p}\left(\hat{A}\right)\subset \sigma_{pp}\left(\hat{A}\right)$— all the eigenvectors |a〉 are in the subspace $H_{pp}$. For finite H (a typical example being the spin-1/2 spinor space, see Appendix A.3), $\sigma \left(\hat{A}\right)=\sigma_{pp}\left(\hat{A}\right)=\sigma_{p}\left(\hat{A}\right)$.

The multiplicity (in quantum mechanics, degeneracy) of an eigenvalue, *a*, is the dimension of its eigenspace. In other words, the degeneracy of any *a* is the number of distinct eigenvectors solving the corresponding eigenvalue equation. We have that $a\in \sigma_{p}\left(\hat{A}\right)$ only if its multiplicity is finite. For instance, for an electron in a constant magnetic field, we have the well-known discrete Landau levels. However, the full wave function has a plane wave component, making the Landau levels infinitely degenerated and the states not in $L^{2}\left(R^{3}\right)$, so formally not true eigenstates in H [86,93].

Lastly, for a self-adjoint $\hat{A}$ (with $\sigma_{p}\left(\hat{A}\right)$ not null), its set of eigenvectors forms the basis for H if

(i) *N* is finite [78];
(ii) *N* is infinite and $\hat{A}$ is compact;
(iii) *N* is infinite and $\hat{A}$ belongs to certain classes of unbounded operators, see [61].

Few remarks are in order here. We recall that an operator $\hat{A}$: H→H is compact *iff* for any bounded sequence $\{|\psi_{n}\rangle \}\in H$, the sequence $\left\{\hat{A}\;|\psi_{n}\rangle \right\}$ has a convergent subsequence in H (see, e.g., [83]). Any compact operator is bounded. For finite *N*, a self-adjoint operator is always compact. A bounded self-adjoint, but not compact, operator is not considered above because it is always unitarily equivalent to a certain multiplication operator [78]. Usually, multiplication operators have just generalized eigenvectors [94] (e.g., delta-functions).

As illustrations, for (ii), we mention a not-too-singular attractive potential (like the hydrogen atom [95]). Then, $\hat{A}$ corresponds to the restriction [96] of the associated Hamiltonian to the subspace of the bound states [85,95]. For (iii), we can cite $\hat{A}$ as the Hamiltonian ${\hat{H}}_{conf}$ of confining potentials, such as the harmonic oscillator [96]. For non-analytic potentials, e.g., the infinite square well, self-adjoint extension techniques can also lead to proper $\hat{A}={\hat{H}}_{conf}$ [97,98,99].

### Appendix A.2. The Structure of the Transformation Matrix Γ

For both $\hat{A}$ and $\hat{B}$ being one of the cases in (i)–(iii) in Appendix A.1 and (n=1,…,N),

(A2) $$\hat{A}\;|a_{n}\rangle =a_{n}\;|a_{n}\rangle ,\;\hat{B}\;|b_{n}\rangle =b_{n}\;|b_{n}\rangle ,$$

and the sets $\left\{|a_{n}\rangle \right\}$ and $\left\{|b_{n}\rangle \right\}$ are normalized bases of the Hilbert space H. In the spin-1/2 Bohm’s version [62,100] of the original EPR construction [19], as well as in the general rigorous definition of EPR states [47] (for infinite-dimensional spaces, see [49,50]), all the bases discussed are orthonormal. This is straightforwardly the case for $\left\{|a_{n}\rangle \right\}$ and $\left\{|b_{n}\rangle \right\}$ if the set of eigenvalues $\left\{a_{n}\right\}$ and $\left\{b_{n}\right\}$ are non-degenerate. On the other hand, eigenvectors belonging to the same eigenvalue in principle do not need to be mutually orthogonal. However, relying on the spectral decomposition theorem for self-adjoint operators on linear Hilbert spaces [78], we can always use the Gram–Schmidt orthogonalization procedure, obtaining a complete orthonormal basis, ONB, for H. Thus, without loss of generality, we can assume $\left\{|a_{n}\rangle \right\}$ and $\left\{|b_{n}\rangle \right\}$ as ONBs.

We now consider two possible situations for the operators $\hat{A}$ and $\hat{B}$ and analyze the structure of the transformation matrix in Equation (4). Thus, we shall rely on the well-known results of the diagonalization of self-adjoint operators, a subject discussed in many quantum mechanics textbooks (for a particularly instructive treatment, see [101]):

- $\hat{A}$ and $\hat{B}$ commute: (1) The operators have common eigenvectors. (2) So, based on which one is diagonal, say $\hat{A}$, $\hat{B}$ is block-diagonal with the distinct blocks having dimensions (all finite, see Appendix A.1) equal to the multiplicity of the different $\hat{A}$ eigenvalues. (3) But from the previous remarks about spectra decomposition, these blocks can always be put in a diagonal form. Therefore, there is at least one basis that simultaneously diagonalizes $\hat{A}$ and $\hat{B}$. As a consequence of (3), Γ in Equation (4) can be reduced to an identity matrix $1_{N}$ of size *N*. In fact, more generally $\Gamma =P_{\pi (N)}$ is a permutation matrix, once in Equation (4), we can have $|a_{n^{\prime \prime }}\rangle ↔|b_{n^{\prime }}\rangle$ for some subsets of indices $n^{\prime \prime }\neq n^{\prime }$. Nonetheless, given that for any permutation matrix, $P_{\pi (N)}^{T}\;P_{\pi (N)}=1_{N}$, a trivial relabeling of one of the basis indices, say $\left\{|a_{n}\rangle \right\}$, turns Γ into $1_{N}$.
- $\hat{A}$ and $\hat{B}$ do not commute: (4) There are no common eigenvectors and, thus, no basis can simultaneously diagonalize $\hat{A}$ and $\hat{B}$. (5) In this way, the matrix Γ may (due to occasional symmetries associated with $\hat{A}$ and $\hat{B}$) or may not display a block diagonal format. However, in either situation, neither Γ itself nor its eventual diagonal blocks can be transformed into identity matrices. If they were, it would imply the existence of mutual eigenvectors, violating condition (4).

An illustration of the above is given next, for spin-1/2 systems.

### Appendix A.3. The Basis Transformation Matrix for Spin-1/2 Systems in Arbitrary Directions

For γ, θ, and ϕ, the first, second, and third Euler angles respectively, the spin-1/2 system component in the direction n≡(sin[θ]cos[ϕ],sin[θ]sin[ϕ],cos[θ]) is represented by the operator (ℏ=1) [102]

(A3) $$\begin{array}{ccc} & & {\hat{S}}_{n}=\frac{1}{2}\left(\begin{array}{cc} \cos[\theta ] & \exp[-i\phi ]\sin[\theta ]\\ \exp[+i\phi ]\sin[\theta ] & -\cos[\theta ] \end{array}\right). \end{array}$$

For the eigenvectors of ${\hat{S}}_{n}$, see [102]. Moreover,

(A4) $$\begin{array}{ccc} \; & \; & |n,+1/2\rangle =\exp\left[-i\frac{\gamma }{2}\right]\left(\begin{array}{c} +\exp[-i\phi /2]\cos[\theta /2]\\ +\exp[+i\phi /2]\sin[\theta /2] \end{array}\right),\\ \; & \; & |n,-1/2\rangle =\exp\left[+i\frac{\gamma }{2}\right]\left(\begin{array}{c} -\exp[-i\phi /2]\sin[\theta /2]\\ +\exp[+i\phi /2]\cos[\theta /2] \end{array}\right). \end{array}$$

Thus, a basis transformation from $n^{\prime }$ to $n^{\prime \prime }$ is written as follows:

(A5) $$|n^{\prime \prime },\pm 1/2\rangle =\Gamma_{+\frac{1}{2},\pm \frac{1}{2}}^{\left(n^{\prime }\to n^{\prime \prime }\right)}\;|n^{\prime },+1/2\rangle +\Gamma_{-\frac{1}{2},\pm \frac{1}{2}}^{\left(n^{\prime }\to n^{\prime \prime }\right)}|n^{\prime },-1/2\rangle ,\;\;\;\;\;\;\;\;$$

leading to a unitary matrix whose elements are (with $\Delta_{\phi }=\left(\phi^{\prime \prime }-\phi^{\prime }\right)/2$, $\Delta_{\theta }^{\left(\pm \right)}=\left(\theta^{\prime \prime }\pm \theta^{\prime }\right)/2$, and $\Delta_{\gamma }^{\left(\pm \right)}=\left(\gamma^{\prime \prime }\pm \gamma^{\prime }\right)/2$)

(A6) $$\begin{array}{ccc} \Gamma_{+\frac{1}{2},+\frac{1}{2}}^{\left(n^{\prime }\to n^{\prime \prime }\right)}\; & = & \exp\left[-i\Delta_{\gamma }^{\left(-\right)}\right]\left(+\cos\left[\Delta_{\phi }\right]\cos\left[\Delta_{\theta }^{\left(-\right)}\right]-i\sin\left[\Delta_{\phi }\right]\cos\left[\Delta_{\theta }^{\left(+\right)}\right]\right),\\ \Gamma_{-\frac{1}{2},+\frac{1}{2}}^{\left(n^{\prime }\to n^{\prime \prime }\right)}\; & = & \exp\left[-i\Delta_{\gamma }^{\left(+\right)}\right]\left(+\cos\left[\Delta_{\phi }\right]\sin\left[\Delta_{\theta }^{\left(-\right)}\right]+i\sin\left[\Delta_{\phi }\right]\sin\left[\Delta_{\theta }^{\left(+\right)}\right]\right),\\ \Gamma_{+\frac{1}{2},-\frac{1}{2}}^{\left(n^{\prime }\to n^{\prime \prime }\right)}\; & = & \exp\left[+i\Delta_{\gamma }^{\left(+\right)}\right]\left(-\cos\left[\Delta_{\phi }\right]\sin\left[\Delta_{\theta }^{\left(-\right)}\right]+i\sin\left[\Delta_{\phi }\right]\sin\left[\Delta_{\theta }^{\left(+\right)}\right]\right),\\ \Gamma_{-\frac{1}{2},-\frac{1}{2}}^{\left(n^{\prime }\to n^{\prime \prime }\right)}\; & = & \exp\left[+i\Delta_{\gamma }^{\left(-\right)}\right]\left(+\cos\left[\Delta_{\phi }\right]\cos\left[\Delta_{\theta }^{\left(-\right)}\right]+i\sin\left[\Delta_{\phi }\right]\cos\left[\Delta_{\theta }^{\left(+\right)}\right]\right). \end{array}$$

From the direct analysis of Equation (A6), one finds that $\Gamma^{\left(n^{\prime }\to n^{\prime \prime }\right)}$ can be reduced to $1_{2}$ (or its associated permutation, Appendix A.2) only for those combinations of the angles $\theta^{\prime \prime }$, $\theta^{\prime }$, $\phi^{\prime \prime }$, $\phi^{\prime }$ corresponding to $n^{\prime \prime }=\pm n^{\prime }$ (and so we can set $\gamma^{\prime \prime }=\gamma^{\prime }=0$). Note this is indeed a consequence of $\left[{\hat{J}}_{n^{\prime \prime }},{\hat{J}}_{n^{\prime }}\right]$ to vanish only if $n^{\prime \prime }=\pm n^{\prime }$ (the minus signal taking into account cases like $\theta^{\prime \prime }=0$ and $\theta^{\prime }=\pi$, for which ${\hat{J}}_{n^{\prime \prime }}={\hat{J}}_{z}$ and ${\hat{J}}_{n^{\prime }}=-{\hat{J}}_{z}$).

Therefore, for $n^{\prime \prime }$ and $n^{\prime }$, which are neither parallel nor anti-parallel, the structure of Γ represents transformations between ONBs associated with non-commuting operators. This exemplifies our general result in the specific case of a spin-1/2 problem.
